# High concentrations of membrane-fed ivermectin are required for substantial lethal and sublethal impacts on *Aedes aegypti*

**DOI:** 10.1186/s13071-020-04512-5

**Published:** 2021-01-06

**Authors:** Max Hadlett, Sanjay C. Nagi, Manas Sarkar, Mark J. I. Paine, David Weetman

**Affiliations:** 1 Department of Vector Biology, Liverpool School of Tropical Medicine, Pembroke Place, Liverpool, L3 5QA UK; 2 Research and Development, Global Innovation-Pest Category, Reckitt Benckiser, Sector 32, Gurgaon, Haryana 122001 India

**Keywords:** Ivermectin, Endectocide, *Aedes*, Arboviruses, IVM

## Abstract

**Background:**

With widespread insecticide resistance in mosquito vectors, there is a pressing need to evaluate alternatives with different modes of action. Blood containing the antihelminthic drug ivermectin has been shown to have lethal and sub-lethal effects on mosquitoes. Almost all work to date has been on *Anopheles* spp., but impacts on other anthropophagic vectors could provide new options for their control, or additional value to anti-malarial ivermectin programmes.

**Methods:**

Using dose-response assays, we evaluated the effects of ivermectin delivered by membrane feeding on daily mortality (up to 14 days post-blood feed) and fecundity of an Indian strain of *Aedes aegypti*.

**Results:**

The 7-day lethal concentration of ivermectin required to kill 50% of adult mosquitoes was calculated to be 178.6 ng/ml (95% confidence intervals 142.3–218.4) for *Ae. aegypti*, which is much higher than that recorded for *Anopheles* spp. in any previous study. In addition, significant effects on fecundity and egg hatch rates were only recorded at high ivermectin concentrations (≥ 250 ng/ul).

**Conclusion:**

Our results suggest that levels of ivermectin present in human blood at current dosing regimes in mass drug administration campaigns, or even those in a recent higher-dose anti-malaria trial, are unlikely to have a substantial impact on *Ae. aegypti*. Moreover, owing to the strong anthropophagy of *Ae. aegypti*, delivery of higher levels of ivermectin in livestock blood is also unlikely to be an effective option for its control. However, other potential toxic impacts of ivermectin metabolites, accumulation in tissues, sublethal effects on behaviour, or antiviral action might increase the efficacy of ivermectin against *Ae. aegypti* and the arboviral diseases it transmits, and require further investigation.
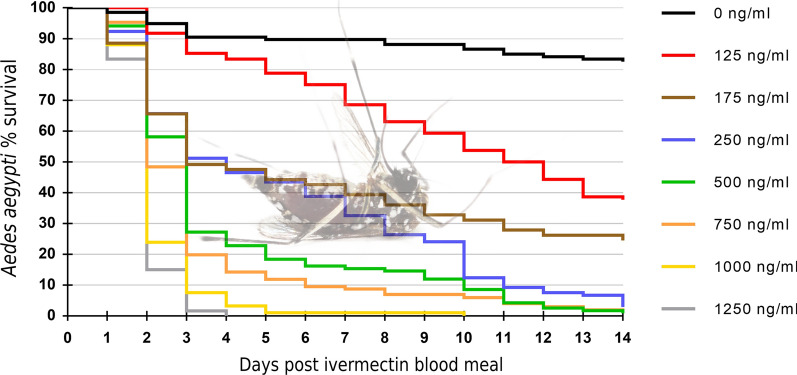

## Background

Escalating insecticide resistance [1, 2], recent high profile arboviral outbreaks [3–6] and the stalling of progress towards malaria elimination [7] highlight the need for improved tools to control mosquito vectors. Compounds with alternative modes of action including new and repurposed insecticides for long-lasting insecticidal nets (LLINs) and indoor residual spraying (IRS) are required and are becoming available now, but there remains an urgent need for tools that can target mosquitoes that bite diurnally or bite and rest outdoors [8, 9].

Ivermectin is a macrocyclic lactone that displays broad spectrum anti-parasitic activity against both endo- and ecto-parasites, including mosquitoes [10–12]. Since the 1990s, the drug has been extensively used in mass drug administration (MDA) campaigns to eliminate lymphatic filariasis and onchocerciasis in sub-Saharan Africa [13, 14]. During these programmes, reductions in entomological indices and the proportion of *Plasmodium*-infective mosquitoes have been observed, compatible with mosquitocidal activity of ivermectin [11, 15, 16]. Consequently, ivermectin administration is currently proposed as a novel strategy to reduce malaria transmission [17–19].

Ivermectin primarily targets the glutamate-gated chloride channel; a different mode of action to insecticides currently available on the public health market [20, 21], and therefore may be effective against mosquitoes resistant to current insecticides. In addition, a crucial difference from LLINs and IRS is that ivermectin has the potential to protect against mosquitoes displaying alternative biting and resting behaviours [22].

The majority of entomological studies conducted using single ivermectin doses have recorded short-lived mosquitocidal effects (< 7 days) [10, 15, 16, 23], but delivery methods capable of sustaining higher venous plasma ivermectin concentrations are now being investigated [24, 25]. In a recent randomised controlled trial, prolonged mosquitocidal effects (> 28 days) were demonstrated when humans were treated with doses of ivermectin over 3 days; importantly, no significant adverse events were reported when the ivermectin was administered alone or co-administered with a standard anti-malarial treatment [26]. A modelling study based on these data further demonstrated the potential of ivermectin-based MDA for malaria control [27]. In addition, uptake of sub-lethal concentrations of ivermectin has been shown to affect mosquito fecundity, locomotion, ability to re-feed [28–32], to inhibit *Plasmodium* sporogony in the vector [33–35] and to adversely affect liver stages of the parasite [36].

In light of the potential for ivermectin MDA to reduce malaria transmission, the majority of research has focused on anopheline vectors. However, in many regions of the tropics, malaria and *Aedes*-transmitted arboviruses are co-endemic [37]. Given the limited resources available to control arboviral diseases in countries where malaria is a primary target, the effects of systemic ivermectin on other vectors should be examined and the opportunity for cross-disease control in integrated vector management programmes evaluated. Earlier studies have suggested that ivermectin displays significantly lower toxicity to the culicines tested than to anophelines [38–41]. However, these studies either did not assess mortality beyond 24 h after blood-feeding, or did not report at which day the lethal dose [e.g. the lethal concentration required to kill 50% (LD_50_) of adult mosquitoes] was calculated. In *Anopheles gambiae*, ivermectin has been shown to affect survival up to 14 days post-blood-feeding [26], and so the potential of delayed mortality in other mosquito vectors also needs evaluation. Additionally, if fecundity is reduced by ivermectin, mosquito populations may be suppressed even if the short-term killing effect is limited. In this study, we conducted laboratory experiments to investigate the effect of imbibed ivermectin on the 14-day survivorship, fecundity, and egg hatch rate of *Aedes aegypti*.

## Methods

### Mosquitoes

A pyrethroid-susceptible *Ae. aegypti* strain, founded from an original collection in Mumbai in 2010, and subsequently maintained at the insectaries of Godrej Consumer Products Limited, Mumbai, India, was used. Adults were maintained on 10% sugar solution at 27 ± 2 °C and 80 ± 10% relative humidity with a 12-h light:12-h dark photoperiod. All mosquitoes were blood-fed at 5–7 days post-emergence.

### Drugs and reagents

A powdered ivermectin formulation, dimethyl sulphoxide (DMSO), and phosphate buffered saline (PBS) were obtained from Sigma-Aldrich (St. Louis, MO). Ivermectin was dissolved in DMSO to a concentration of 10 mg/ml and refrigerated overnight at 4 °C. The 10 mg/ml ivermectin stock was then serially diluted into PBS to create two working stock solutions: 1 mg/ml and 0.1 mg/ml. These working stock solutions were diluted further in PBS to achieve final concentrations that were ten times higher than the concentrations required in blood meals (10× stocks). The control stock solution contained PBS with DMSO at a concentration equivalent to the highest concentration of ivermectin working stock. All stocks were stored in sealed conical flasks and refrigerated until required at 4 °C. Final stocks were diluted to 1× into blood as required.

### Blood-feeding and ivermectin administration

Defibrinated goat blood sourced from a local abattoir was collected fresh every 2 days and used for all blood feeds. Prior to blood-feeding, mosquitoes were starved of sugar for 6 h. Five- to 7-day-old female mosquitoes were held in 30-cm × 30-cm cages and offered blood using a Hemotek feeder (Discovery Workshops, Accrington, UK) (placed on the upper surface) covered with collagen membrane and heated to 37 °C. Mosquitoes were given the opportunity to feed for approximately 30 min. Blood contained ivermectin at the following concentrations: 0; 125; 175; 250; 500; 750; 1000; and 1250 ng/ml, which were chosen based on previous studies in culicines.

### Mosquito survival

After experimental blood-feeding, fully engorged females were removed from each cage using a mouth aspirator and placed into empty paper test cups (*n* = 5 mosquitoes in each). Mosquitoes were provided with cotton wool soaked in 10% sugar solution. Mortality was recorded every 24 h for 14 days post-blood-feeding, with dead mosquitoes removed daily. Owing to logistical constraints, more experimental replicates were performed for the 0, 125, 250, 750 and 1000 ng/ml groups than for the 175, 500, and 1250 ng/ml groups, but a minimum of 60 blood-fed mosquitoes were available per experimental concentration (mean ± SD = 97.6 ± 30.0).

### Egg production

The impact of blood-fed ivermectin on the number of eggs produced from surviving females was assessed using the following methodology: 48 h post-blood-feeding, surviving mosquitoes were carefully removed from the holding cups using a mouth aspirator and placed individually into fresh oviposition cups. An upturned plastic bottle cap filled with 4 ml of water and a 2-cm × 2-cm square of damp Whatman filter paper was provided as an egg-laying substrate. Mosquitoes were provided with cotton wool soaked in 10% sugar solution on top of the mesh used to seal the cup. Seventy-two hours later (5 days post-blood feed), bottle caps were removed and any eggs were counted using a stereoscopic dissection microscope at ×2 magnification. After counting, all eggs were left on filter paper to dry at ambient room temperature for a further 48 h, prior to determination of hatching rates.

### Egg hatch rate

To analyse whether a sub-lethal dose of ivermectin affected the hatch rate of eggs laid by surviving adults, eggs on filter paper were submerged in 200 ml of filtered water inside 250-ml plastic pots. A sample of 200 eggs randomly selected from the control (0 ng/ml) and four of the seven treatment groups (125, 175, 250 and 500 ng/ml) were taken for analysis. No eggs were taken from three high concentration treatment groups (750, 1000 and 1250 ng/ml) either because an insufficient number were produced, or because mosquitoes died before oviposition. Ninety-six hours after submersion, larvae from each pot were transferred into new water-filled pots using a mesh net, and the number emerging from each pot was recorded.

### Statistical analysis

The LC_50_ of adult mosquitoes was calculated using log-probit regression analysis in SPSS version 21 (IBM SPSS). Kaplan-Meier survival analysis followed by a log-rank (Mantel-Cox) test using the survival package in R version 3.6.3 was used to assess the effect of ivermectin on survival of adult females. Egg production data were not normally distributed (Shapiro–Wilk test), therefore a Kruskal–Wallis non-parametric ANOVA was employed to examine variation among concentrations. To assess whether there was any significant difference in the number of eggs produced per surviving female between ivermectin test groups and the negative control group, a post-hoc Dunn’s multiple comparison test was used. The effect of ivermectin on egg hatch rate was assessed by comparing the hatch rate between the negative control group and each individual ivermectin group using Fisher’s exact tests. Egg production and egg hatch rate data were analysed using GraphPad Prism 7.03.

## Results

**Fig. 1:**
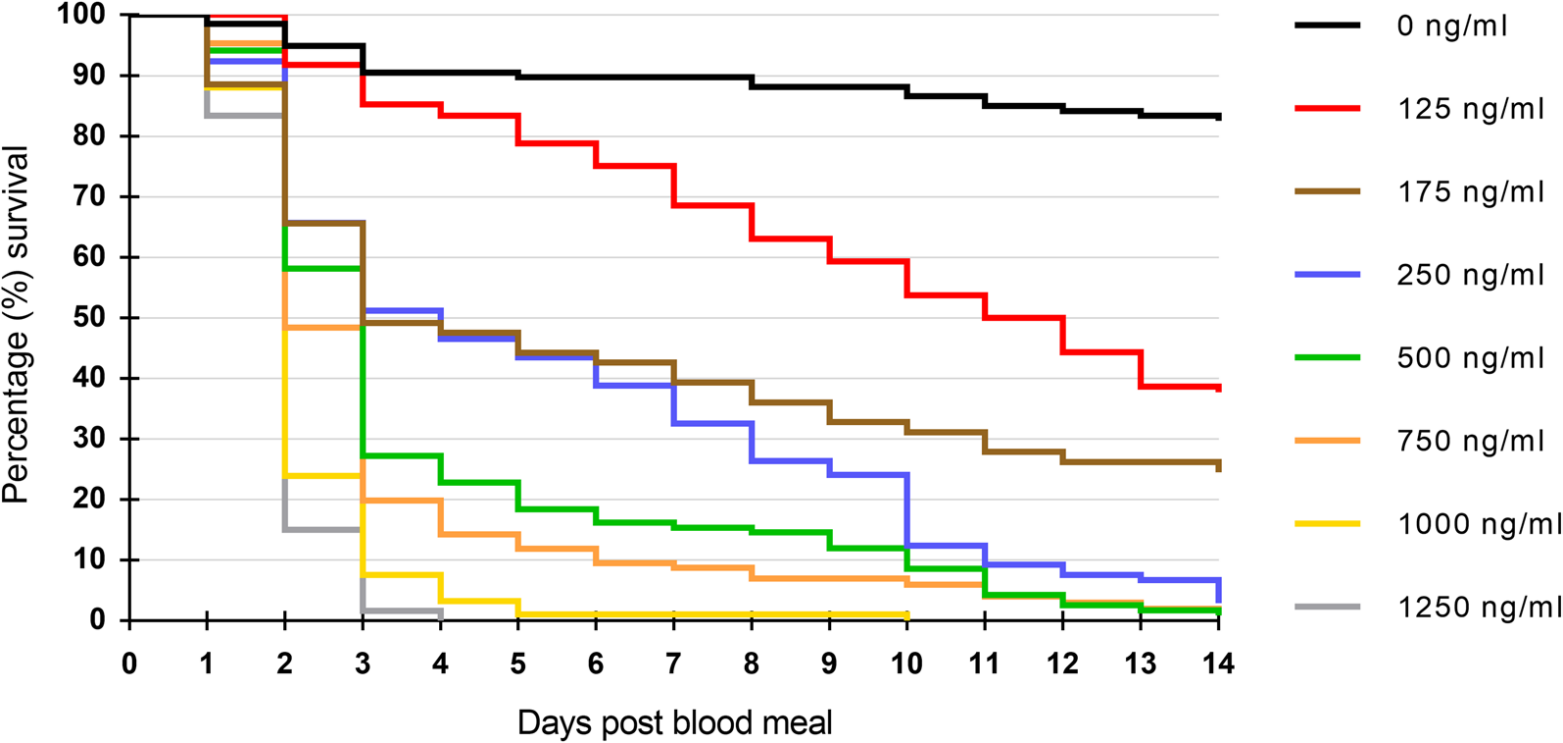
Fourteen-day Kaplan-Meier survival curves showing the daily survival of *Aedes aegypti* following ingestion of ivermectin at varying concentrations

A total of 781 *Ae. aegypti* took blood meals and were used to assess the oral toxicity of blood-fed ivermectin. Kaplan-Meier 14-day survival curves were produced for the daily survival of mosquitoes following the ingestion of ivermectin at concentrations ranging from 0 to 1250 ng/ml (Fig. 1). Survival was high in the zero-ivermectin control throughout, but ivermectin ingestion significantly reduced the survival of *Ae. aegypti* mosquitoes (log-rank test,* df* = 7,* P* < 2e − 16). This was true for all ivermectin concentrations tested, when compared individually against the control arm and adjusting* p*-values with a Bonferroni correction for multiple testing (log-rank test,* df* = 1, all* P* ≤ 1e − 09). Survival was quite high at all concentrations relative to the control group, until the second day after blood-feeding when substantial mortality occurred in a concentration-dependent manner, and by day 4 all females had died at the highest concentration (Fig. 1).

**Fig. 2:**
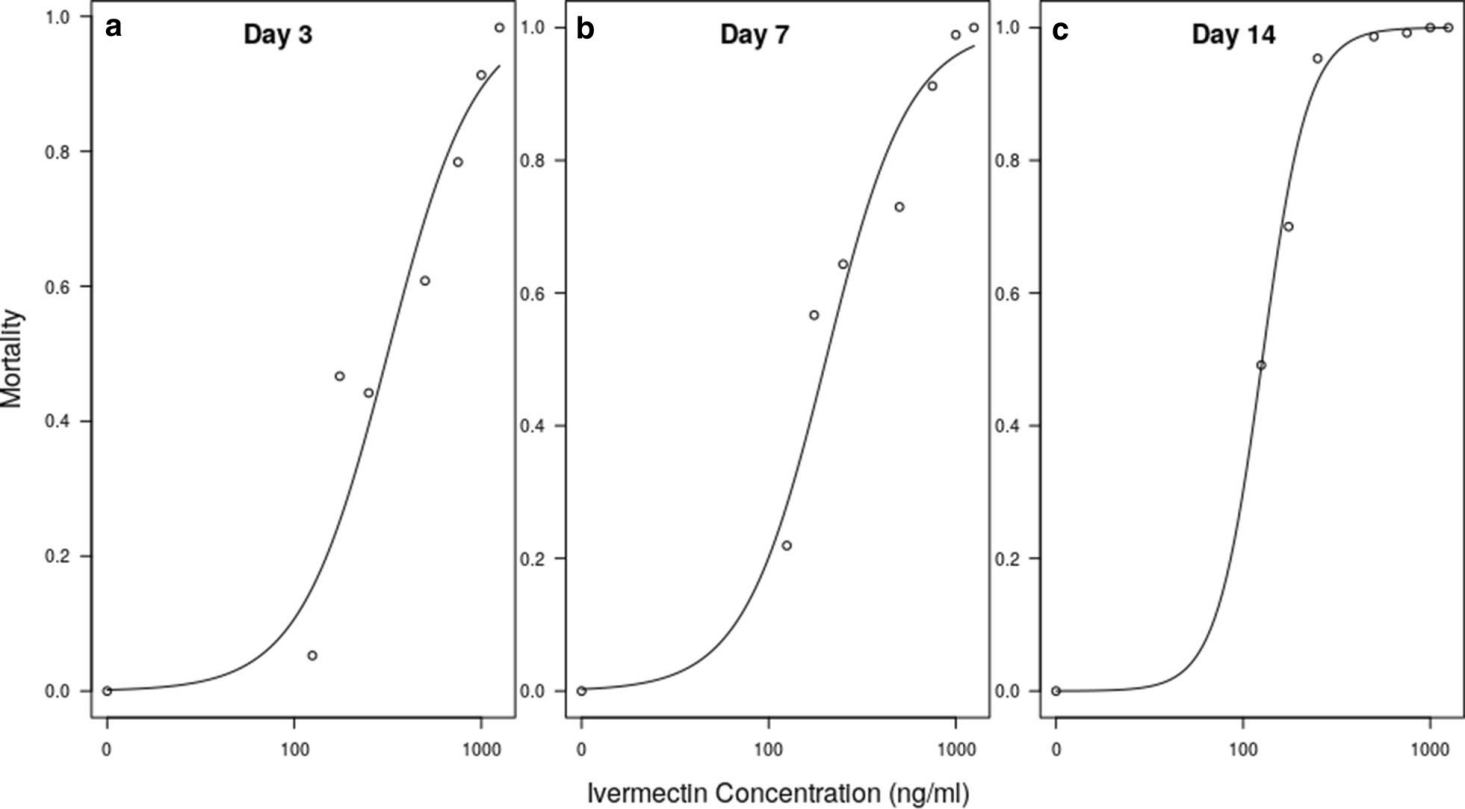
Dose-response curves of membrane-fed ivermectin against *Ae. aegypti* at **a** day 3, **b** day 7, **c** day 14

Figure 2 shows dose-response curves at days 3, 7 and 14, and Table 1 shows the resulting estimates for LC_20_, LC_50_ and LC_90_. The LC_50_ of female *Ae. aegypti* was dependent on the time after ingestion, with a 35% lower LC_50_ on day 7 than on day 3, and a 71% lower estimate on day 14, demonstrating the delayed mortality effect of ivermectin.

**Table 1:** Lethal concentrations (*LC*) of ivermectin for *Aedes aegypti* at days 3, 7 and 14 post-blood-feeding, calculated using probit analysis

LC (%)	Ivermectin concentration (ng/ml) (95% confidence interval)		
	Day 3	Day 7	Day 14
20	125.5 (94.1–157.5)	81.6 (58.6–105.6)	36.3 (23.2–51.3)
50	274.7 (225.6–330.1)	178.6 (142.3–218.4)	79.5 (57.1–104.9)
90	905.4 (723.5–1202.3)	588.8 (473.9–765.9)	262.1 (203.2–344.2)

**Fig. 3:**
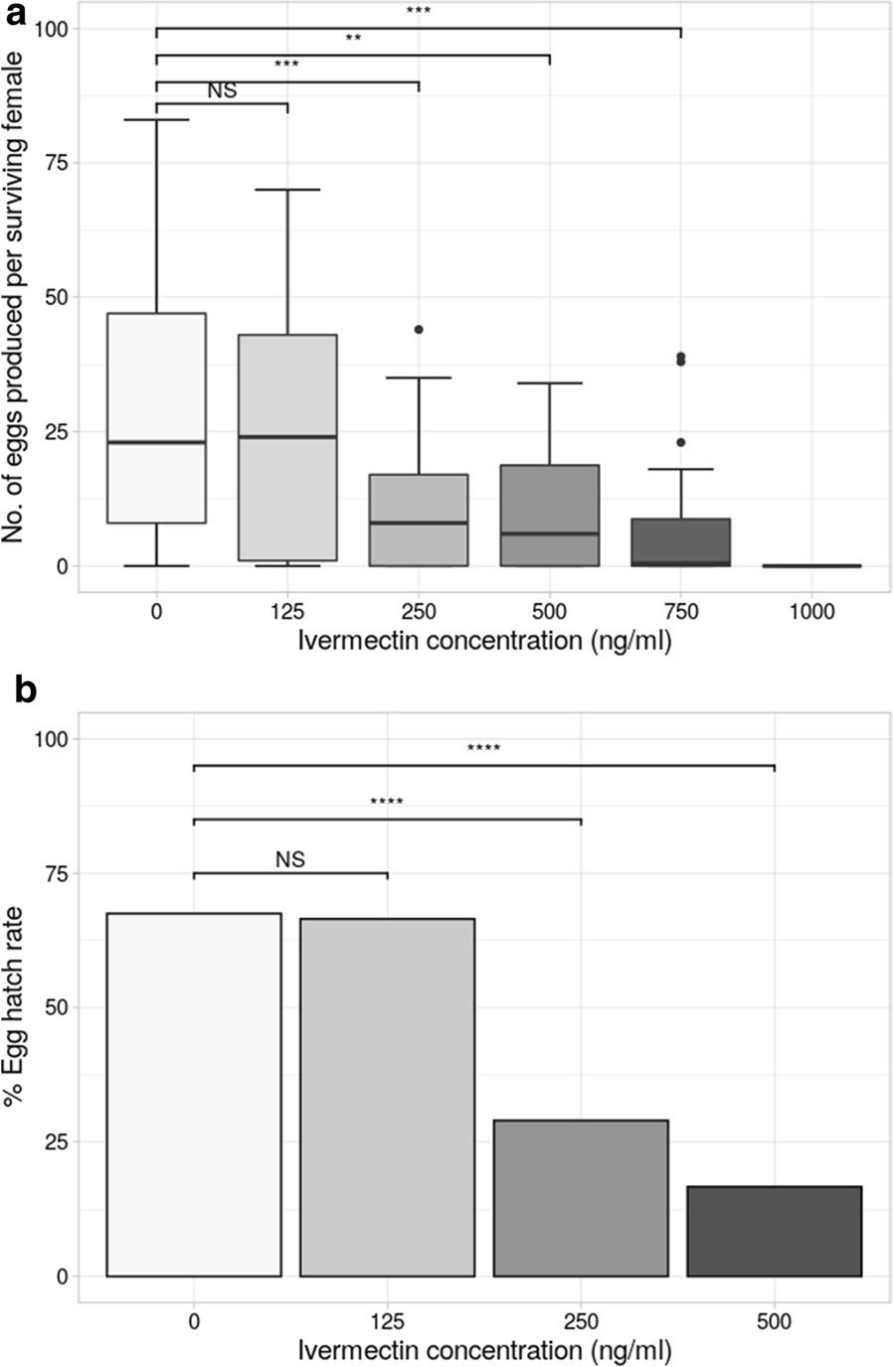
**a**, **b** Reproductive output of *Ae. aegypti* following ivermectin ingestion. **a** Average number of eggs produced per surviving female mosquito. **b** Hatch rate (%) of eggs produced by surviving females.* Asterisks* represent the level of statistical difference from the control group (0 ng/ml); * *P* < 0.05, ** *P* < 0.01, *** *P* < 0.001, **** *P* < 0.0001; groups that did not differ significantly from each other are grouped by a* horizontal line above the bars*

Ivermectin-feeding exerted a significant impact on the fecundity of surviving female *Ae. aegypti* (Fig. 3a). Whilst fecundity at 125 ng/ml was similar to that of the unexposed control group, each higher concentration significantly reduced the mean number of eggs produced, with fecundity following the highest dose reduced 20 times compared to the control group (Table 2). In addition to its effect on fecundity, ivermectin also reduced the egg hatch rate of surviving *Ae. aegypti* females (Fig. 3b). Similar to effects on fecundity, exposure to 125 ng/ml had little impact on hatch rate, but this was significantly reduced at the higher concentrations (for which sufficient egg numbers were available), with an approximately fourfold reduction at 500 ng/ml (Table 2).

**Table 2:** Fecundity of *Ae. aegypti* following ingestion of ivermectin at varying concentrations

Ivermectin (ng/ml)	No. of mosquitoes fed	No. of surviving females on day 3 (% survival)	Mean no. of eggs laid/surviving female ± SEM	% Hatch rate (no. of hatched larvae/no. of eggs submerged)
0	67	60 (89.6)	28.4 ± 2.9	69 (138/200)
125	54	49 (90.7)	23.5 ± 2.9	66.5 (133/200)
250	69	44 (63.7)	10.8 ± 1.7	29 (58/200)
500	74	26 (35.1)	10.0 ± 2.3	16.7 (25/150)
750	65	18 (27.7)	7.8 ± 3.1	–^a^
1000	32	3 (9.4)	0 ± 0	–^a^

*SEM* standard error of the mean

^a^Hatch rate was not calculated for eggs of mosquitoes exposed to ≥ 750 ng/ml

## Discussion

Ivermectin holds significant promise to expand the current vector control toolbox. Alongside its excellent human safety profile and lack of impact on non-haematophagous insects, it has the potential to impact disease transmission occurring outdoors, and a novel mode of action which may circumvent insecticide resistance. Though it has proven effective at killing *Anopheles* mosquitoes, little research to date has focused on its effect on other disease vectors. In this study we assessed the effect of ivermectin on survivorship, fecundity and egg hatch rate in *Ae. aegypti*.

Survival of *Ae. aegypti* declined significantly when it membrane-fed on blood containing ivermectin, at all concentrations tested. In concordance with previous studies, the lethal dose for ivermectin was relatively high, with a 7-day LC_50_ of 178.6 ng/ml. This is in agreement with a previous study which estimated 7-day LC_50_s for multiple *Ae. aegypti* strains ranging from 187.17 to 576.43 ng/ml [39]. For comparison, recent studies have demonstrated 7-day LC_50_ doses for *Anopheles* spp. ranging between 3.35 and 55.6 ng/ml [23, 34, 42–44].

In MDA campaigns to control filarial disease, ivermectin is typically delivered as a single dose at approximately 150–200 µg/kg [45]. These single doses have been shown to cause mosquitocidal effects for only short periods of less than 7 days [10, 15, 16, 44]. Ivermectin has a half-life of approximately 18 h, and at doses of 150–200 µg/kg typical peak venous plasma concentrations are in the range of 10–70 ng/ml [46]. A recent randomised controlled trial in Kenya demonstrated prolonged mosquitocidal effects of ivermectin for up to 28 days after administration against *An. gambiae*, through 3-day dosing of 300 and 600 µg/kg [26]. In pharmacokinetic data from the same trial, a median maximum recorded concentration (*C*_max_) of ivermectin of 105.2 ng/ml was found in venous blood at the highest dose, with the time to reach *C*_max_ of approximately 4 h [47]. That peak ivermectin concentration surpasses the 7-day LC_20_ for the *Ae. aegypti* strain used in the present study. However, a potential caveat is that there could be an additional impact from metabolites of ivermectin, which are present in human blood days after administration, and which are toxic to mosquitoes. This was suggested by the observation that the duration of mortality observed in *An. gambiae* was far longer than that projected by pharmacokinetic models [26, 45]; however, subsequent analysis showed it was not necessary to invoke this mechanism [47]. Whilst it seems clear that the duration of efficacy is likely to be much shorter in *Ae. aegypti* relative to *Anopheles* spp. for any given dose, the precise impact of ivermectin in human venous blood on *Ae. aegypti* requires further investigation.

Another important factor is the capacity of ivermectin to exhibit a range of sub-lethal effects in mosquitoes, meaning that it could still impact disease transmission through reductions in mosquito fecundity, egg hatching, or ability to re-feed. We found a significant impact of ivermectin in reducing fecundity, and also egg hatch rates, at concentrations ≥ 250 ng/ml (Fig. 3). At the lowest concentration tested (125 ng/ml), there was no difference in the mean number of eggs laid, nor in the egg hatch rate. This concentration is higher than that found in human blood even at a high dosing regime [48], and thus it seems unlikely that ivermectin would have a measurable impact on *Ae. aegypti* via sub-lethal impacts. However, it is important to note both the caveat above (potentially toxic metabolites), and also that, in *Ae. aegypti*, effects on fitness-related behaviours such as the ability to re-feed have yet to be explored.

Ivermectin has also been proposed as a veterinary endectocide [31, 49, 50]. Deployed in this manner, much higher ivermectin concentrations could potentially be reached; however, *Ae. aegypti* typically displays limited zoophagic behaviour and effects are likely to be limited accordingly. However, an impact remains possible for other important arbovirus vectors that display more plasticity in feeding behaviour, such as *Aedes albopictus* [51–54].

A limitation of the study was that the membrane feeds were conducted solely on a single relatively homogeneous laboratory strain of *Ae. aegypti*, which may now differ substantially from the field mosquitoes from which it was originally established. Additionally, ivermectin is currently proposed as a control tool primarily in sub-Saharan Africa; compared to Asian populations, African *Ae. aegypti* are known to differ genetically and exhibit phenotypic differences, such as in susceptibility to arboviral infections, or mechanisms of resistance to insecticides [37, 55, 56]. Another possible limitation is that ivermectin was delivered systemically via Hemotek membrane feeding, and this method of delivery may inaccurately represent ivermectin uptake compared to direct blood-feeding on humans. For example, it has been suggested that ivermectin, as a lipophilic compound, may accumulate in dermal and adipose tissue and therefore reach higher concentrations than that found in venous plasma [57]. As mosquitoes imbibe blood from subdermal capillaries, they may ingest concentrations of ivermectin higher than those found in venous plasma [57]. Despite this concern, a recent study, nested within a randomised controlled trial, found similar mosquitocidal effects of ivermectin when comparing membrane feeding to direct skin feeding [43].

Finally, it remains possible that arbovirus replication and transmission could be directly inhibited by ivermectin. For example, a recent study demonstrated the antiviral activity of ivermectin against dengue serotype 2 when blood-fed to *Ae. albopictus* [58]. Additionally, *in vitro* enzymatic assays have shown ivermectin to inhibit the replication of other flaviviruses, such as yellow fever virus [59]. Whether sub-lethal concentrations of ivermectin could affect vector competence for arboviruses in the mosquito host should be explored further.

In conclusion, we have demonstrated through membrane feeding experiments that high concentrations of ivermectin are required to induce mortality and affect the fecundity of *Ae. aegypti.* Therefore, potential control strategies employing ivermectin MDA are unlikely to have a substantial impact on *Aedes*-transmitted arboviral disease. However, further research is necessary to determine if ivermectin exhibits additional effects such as sub-lethality and antiviral activity.

## Data Availability

Data are available from the corresponding author on request.
